# The PD-1 with PD-L1 Axis Is Pertinent with the Immune Modulation of Atrial Fibrillation by Regulating T Cell Excitation and Promoting the Secretion of Inflammatory Factors

**DOI:** 10.1155/2022/3647817

**Published:** 2022-05-12

**Authors:** Guodong Chang, Yingwei Chen, Zichang Liu, Yong Wang, Wenkun Ge, Yongan Kang, Shuling Guo

**Affiliations:** ^1^Department of Cardiology, The First People's Hospital of Shangqiu, China; ^2^Department of Cardiology, The First Affiliated Hospital of Zhengzhou University, China; ^3^Department of Cardiology, Xuchang Central Hospital, China

## Abstract

**Objective:**

To analyze the role of PD-1/PD-L1 signaling pathway in regulating T cell activation and secretion of proinflammatory factors in atrial fibrillation.

**Methods:**

Forty-five patients with atrial fibrillation admitted to the cardiology department of our hospital from July 2019 to March 2021 were selected to be included in the atrial fibrillation group, and another 45 healthy volunteers were selected as the control group to compare the changes of T cell CD69 and human leukocyte antigen-DR (HLA-DR) expression in the peripheral blood of the two study groups; compare the changes of programmed death factor-1 on CD4+ and CD8+ lymphocytes in the peripheral blood of the two groups (PD-1) expression changes and PD-L1 and PD-L2 expression changes on peripheral blood myeloid dendritic cells (mDCs) cells; compare the changes of interleukin-2, interleukin-6, interleukin-10, and interleukin-17A (IL-2, IL-6, IL-10, and IL-17), tumor necrosis factor (TNF), and interferon gamma (IFN-*γ*) concentrations on peripheral blood inflammatory factors in the two groups; and isolate the two groups of peripheral blood mDCs cells; *α* interferon upregulated PD-L1 expression in the cells and analyzed the effect of PD-L1 expression on the ability of mDCs to stimulate T cells to secrete cytokines.

**Results:**

The positive expression rates of CD69 and HLA-DR on peripheral blood CD3+ T lymphocytes were significantly higher in patients in the atrial fibrillation group than in the control group, and the differences were statistically significant (*P* < 0.01). The positive expression rate of PD-1 on CD4+ lymphocytes was significantly lower in patients in the atrial fibrillation group than in the control group (*P* < 0.01). There was no statistically significant difference between the two groups in terms of PD-1 positive expression rate on CD8+ lymphocytes (*P* > 0.05). The positive expression rate of PD-L1 on mDCs cells was significantly lower in patients in the atrial fibrillation group than in the control group (*P* < 0.01), and there was no statistically significant difference between the two groups in the positive expression rate of PD-L2 on mDCs cells, PD-L1, and PD-L2 on CD4+ and CD8+ T cells (*P* > 0.05). The concentrations of IL-2, IL-6, IL-10, and IFN-*γ* in peripheral blood were significantly higher in patients in the atrial fibrillation group than in the control group (*P* < 0.05), and there was no statistically significant difference in the comparison of IL-17A and TNF concentrations in peripheral blood between the two groups (*P* > 0.05). In the atrial fibrillation group, the ability of mDCs to stimulate T cells to secrete IL-2 and IFN-*γ* was significantly higher, and the ability to secrete IL-10 was significantly lower compared with the control group (P < 0.05). After *α* interferon upregulated PD-L1 expression in cells, the ability of mDCs to stimulate T cells to secrete IL-2, IL-10, and IFN-*γ* cytokines was reversed in patients in the atrial fibrillation group, and the differences compared with the control group were not statistically significant (*P* > 0.05).

**Conclusion:**

PD-1/PD-L1 signaling pathway may play an immunomodulatory role in the pathogenesis of atrial fibrillation by promoting increased secretion of inflammatory factors through regulating T cell activation.

## 1. Introduction

Atrial fibrillation (AF) is a frequent clinical persistent arrhythmia, and the occurrence is closely related to hypertension, coronary artery disease, and heart failure. The incidence of AF in adults is about 0.3% to 0.4%, and it increases with age, and the incidence of people over 75 years old can reach 10%. Relevant data show that AF is prone to thrombosis leading to stroke, especially thromboembolic events. The risk of death increases by 50% to 90%, and it has become one of the major cardiovascular diseases affecting public health [1, 2]. At present, the pathophysiological mechanism of AF has not been fully clarified, and the relation between inflammation and AF has aroused widespread concern in recent years.

In the process of AF, the atrial plasma and cells in the center may trigger membrane potential pulsation, change atrial electrophysiological activities, and participate in atrial remodeling during the occurrence of AF. In addition, the inflammatory factor C-reactive protein can lead to degeneration, and the necrosis of cardiomyocytes during the inflammatory reaction promotes atrial remodeling during the coming up of AF and induces persistent AF. Therefore, the inflammatory response is considered to be related to the happen and evolution of AF [3, 4]. Many immune cells are involved in the inflammatory response. The immune mechanism is the most important defense mechanism in the body, and it exerts vital effect on the expression of cytokines, immune regulation, and intracellular signaling pathways in the cardiovascular system.

Programmed cell death-1 (PD-1) together with PD-L1 (the ligand of PD-1) are regulatory molecules with negative costimulatory signals and evoke vital function in modulating various aspects of the immune system, such as infection immunity, autoimmunity, and tumor immunity [5, 6]. Among them, the PD-1 with PD-L1 axis is highly expressed in the cardiovascular system, but whether this pathway is related to the pathological progress of AF and whether it is related to inflammatory response is not yet fully understood.

This research took patients with AF as the research object and aimed to analyze the character of PD-1/PD-L1 in regulating T cell excitation and the secretion of proinflammatory cytokines in AF, which could be contributed to the development of AF treatment.

## 2. Materials and Methods

### 2.1. General Data

**Figure 1:**
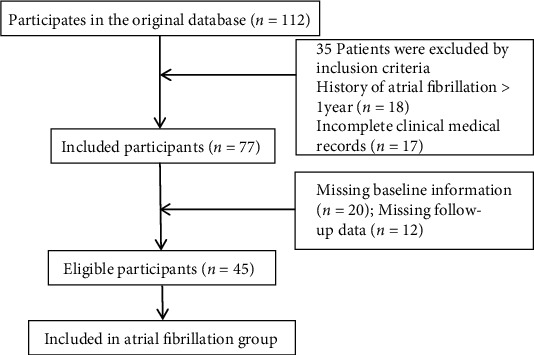
Flow chart of data selection.

45 patients with AF recorded to the Department of Cardiology of our hospital from July 2015 to March 2021 were selected and included in the AF group. This research was authorized by the ethics committee of hospital. The inclusion criteria are as follows: (1) all patients satisfy the clinical diagnostic criteria for AF [7]; (2) history of AF < 1 year; (3) successful drug or electrical cardioversion and maintain sinus rhythm for more than one month; (4) patients without left atrial appendage thrombosis; (5) patients with complete clinical medical records; and (6) all patients and their families gave informed consent to take part in this study. The exclusion criteria are as follows: (1) patients who have severe hepatic or nephritic damage and other vital diseases in organ; (2) patients with recent infectious diseases; (3) patients who have acute myocardial infarction within one month; (4) patients with immune system diseases, including hyperthyroid heart disease and Cushing syndrome; (5) those who had cerebral infarction or cerebral hemorrhage within half a year; and (6) those who received anti-inflammatory or immunotherapy recently. Another 45 healthy volunteers were chosen and absorbed in the control group, with no history of arrhythmia after routine clinical examination. The flow chart of data selection was shown in Figure 1.

### 2.2. Experimental Methods

#### 2.2.1. Detection of the First Signal Molecule of T Cell Excitation [8]

On the second day after admission, 5 ml of fasting venous blood in the morning was drawn, and the supernatant was collected after centrifugation. Flow cytometry was applied to detect the expression levels of CD69 and human leukocyte antigen-DR (HLA-DR) in external blood CD3^+^ T lymphocytes of the two groups of subjects. Heparin sodium anticoagulant venous blood from the two groups of subjects was collected and added into flow test tubes, which were placed at room temperature for 6 h. Fluorescently labeled flow antibodies were added into the tubes, and 2 ml red blood cell lysate was added, which stood for 15 min in dark, and the supernatant was discarded after centrifugation. 1 ml of PBS was added to the test tubes, respectively, and the supernatant was abandoned after centrifugation; Then, PBS was added again and mixed, and the contents of CD69 and HLA-DR were detected by four-color flow cytometer.

#### 2.2.2. The Content of PD-1 and PD-L1

On the second day after admission, 5 ml of fasting venous blood in the morning was drawn, and the supernatant was collected after centrifugation. Flow cytometry was employed to observe the changes of PD-1 content in CD4^+^ and CD8^+^ T lymphocytes and the changes of PD-L1 and PD-L2 expression in myeloid DC (mDCs), CD4^+^, and CD8^+^ T in external blood of the subjects; the steps were the same as above.

#### 2.2.3. Inflammatory Factor Expression [9]

On the second day after admission, 5 ml of fasting venous blood in the morning was drawn, and the supernatant was collected after centrifugation. ELISA assay was applied to measure the external blood inflammatory factors IL-2, IL-6, IL-10, IL-17A, TNF, and IFN-*γ* concentration: add 50 *μ*L of buffer and standard, sample and control substance to each well, add 50 *μ*L of detection antibody, blanking the plate, shake gently and hatch at 20°C ± 5°C for 2 hours, and then wash the plate again after aspirating. After the plate was closed, streptavidin marked with horseradish peroxidase was appended and cultured at 20°C ± 5°C for 45 min. After the liquid was removed, 100 *μ*l TMB was added to develop color. Cultured at dark environment for 20 min, stop solution was appended to terminate color reaction, and OD value was detected at 450 nm.

#### 2.2.4. The Function of PD-1 Together with PD-L1 on the Immune Reaction of Patients with AF [10]

mDCs, CD4^+^, and CD8^+^ T were derived from external blood of the two groups of subjects, and CD4^+^ or CD8^+^ T (isolated from healthy subjects) were mixed with mDCs of the AF group and the control group, and allograft lymph node cell proliferation test was conducted to measure the effect on the secretion of inflammatory factors. IFN-*α* was used to enhance PD-L1 content on mDCs. Besides, the influence of PD-L1 level on the ability of mDCs to stimulate T cells to secrete cytokines was analyzed

### 2.3. Statistical Method

SPSS21.0 software package was employed for data analysis in this study. Detection results were expressed as (*x* ± *s*). Mean value in two groups was compared by independent sample *t*-test. Besides, one-way ANOVA was applied to compare the mean among multiple groups. Counting data were expressed as [*N* (%)]. *χ*^2^ test was taken to contrast the data in two groups. Multivariate COX analysis was used for the determination of the relationship between peripheral blood inflammatory factors and stroke in patients with atrial fibrillation. Kaplan-Meier curve was employed to analyze the risk of stroke in patients with atrial fibrillation with different SIS levels. *P* < 0.05 was viewed as significant.

## 3. Results

### 3.1. Comparison of General Data

No significant difference was observed in general information in AF group and control group (*P* > 0.05). See Table 1.

### 3.2. Changes of CD69 and HLA-DR Expression on External Blood CD3+ T Lymphocytes

The positive expression rates of CD69 together with HLA-DR in CD3^+^ T lymphocytes in external blood in the AF group were markedly larger than the rate in control (*P* < 0.01). See Table 2.

### 3.3. Changes of PD-1 Expression on External Blood CD4^+^ and CD8^+^ T Lymphocytes

The positive expression rate of PD-1 on CD4^+^ lymphocytes in the AF group was memorably fewer than the rate in control (*P* < 0.01). Indifference was observed in the positive expression rate of PD-1 on CD8^+^ lymphocytes in AF and control groups (*P* > 0.05). The results were indicated in Table 3.

### 3.4. Changes of PD-L1 and PD-L2 Content on External Blood mDCs, CD4^+^, and CD8^+^ T Cells

The positive rate of PD-L1 in mDCs cells in AF was prominently lower than the rate in control (*P* < 0.05), and no statistical significance was examined in the positive rate of PD-L2 on mDCs cells and the PD-L1 and PD-L2 levels in cells of CD4^+^ and CD8^+^ T (*P* > 0.05). The results were displayed in Table 4.

### 3.5. Comparison of Expression of Cytokines in External Blood

The concentrations of IL-2, IL-10, IL-6, and IFN-*γ* in external blood in AF group were notably larger than the contents in control (*P* < 0.01), but indifference was detected in the contents of IL-17A and TNF (*P* > 0.05), as shown in Table 5.

### 3.6. Effects of PD-1 and PD-L1 Axis on Immune Function of T Lymphocytes

Compared to the control, the competence of mDCs to stimulate T cells to secrete IL-2 and IFN-*γ* in the AF group was signally elevated, and the ability to secrete IL-10 was prominently descended (*P* < 0.05). After the content of PD-L1 was promoted by IFN-*α*, the capacity of mDCs to stimulate T cells to secrete IL-2, IL-10, and IFN-*γ* cytokines in patients with AF was reversed (*P* > 0.05). See Table 6.

### 3.7. Multivariate COX Analysis of the Relationship between External Blood Inflammatory Factors and Stroke

Multivariate COX stepwise analysis was performed on external blood inflammatory cytokines. The findings identified that IL-2, IL-6, IL-10, and IFN-*γ* were risk factors for stroke risk in AF patients (*P* < 0.05). See Table 7.

### 3.8. Relationship between External Blood Systemic Inflammation Score and Stroke Endpoints

**Figure 2:**
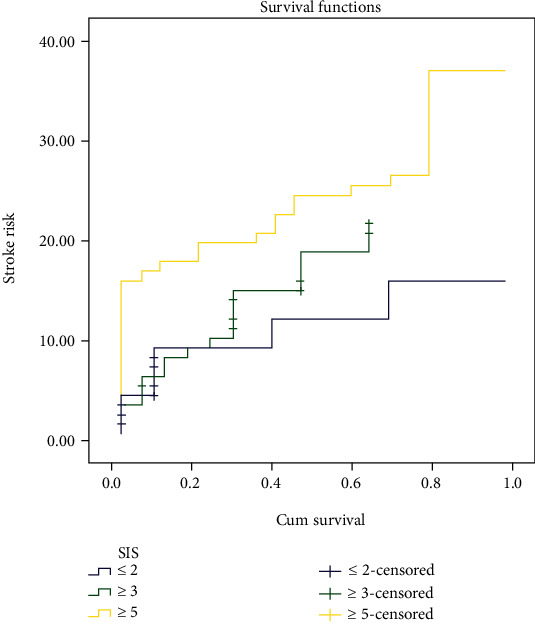
Kaplan-Meier curve analysis of the risk of stroke in patients with AF with different SIS levels.

The statistically significant inflammatory factors in Table 7 were included in the establishment of the systemic inflammation score (SIS). According to change of ILs and IFN-*γ* levels, different values were assigned: 1 for all increased and 0 for all decreased. The level of each inflammation-related factor was integrated into the SIS score (0-6). Univariate analysis showed that notable difference in SIS was measured between the two groups with and without stroke (*P* < 0.05). Multivariate COX analysis displayed that patients with high SIS scores had an increased risk of stroke. The KM survival curve chart showed that the stroke risk of high-level SIS was higher than that of low-level and intermediate-level patients (*P* < 0.05). See Tables 8 and 9 and Figure 2.

## 4. Discussion

With the rapid development of health and medical services in recent years, the treatment of AF has been optimized, but with the increase of age, the hospitalization rate and fatality rate are still increasing year by year, posing a serious threat to human health. AF is considered to be a pathological process influenced by multiple factors, and its complex pathogenesis has not been elucidated so far. It is generally believed that immune inflammatory response and oxidative stress are the main factors leading to the occurrence of AF [11]. Immune response and immune regulation are the central links of inflammatory response. T lymphocytes and their subsets are important factors in the body's immunity, participating in various proinflammatory and anti-inflammatory processes, which in turn affect the progression and maintenance of AF. Relevant data indicate that, the number of CD3^+^ cells in atrial adipose tissue in patients with AF is significantly increased, and the severity of atrial may lead to the transformation of AF types. However, whether T lymphocyte subsets are responsible for the structural changes in the heart in patients with AF has not been established [12, 13]. In order to determine whether immune excitation and multiplication of T lymphocytes exist in external blood of patients with AF, this study detected the content of CD69 and HLA-DR in external blood of patients with AF and robust people and discovered that the positive rate of CD69 associate with HLA-DR in CD3^+^ T lymphocytes in the external blood of AF was prominently larger than the rate in control. The above findings identify that the increased content of CD69 with HLA-DR in CD3^+^ T may be related to the occurrence and progress of AF, and there may be immune excitation of T lymphocytes in external blood of patients with AF. CD69 and HLA-DR are the main signal molecules of T cell excitation, and the increase of their level on immune cells indicates the excitation of the immune cells. It has been proved that CD69 and HLA-DR evoke vital function in many resist diseases, including rheumatoid arthritis and systemic lupus erythematosus [14].

Affiliates of the B7/CD18 family exert a vital function on modulating the balance in T cell excitation and inhibition. The PD-1 and PD-L1 axis is a momentous member of this family, which can induce and maintain immune tolerance in external tissues under normal conditions. It has a positive role in preventing excessive tissue inflammation and also exerts a vital regulatory role in miscellaneous of infectious and autoimmune diseases [15, 16]. After PD-1 binds to the ligand PD-L, they involved in the excitation, growth, and apoptosis of T cells and restrain the immunological response mediated by T cells. Relevant data show that in terms of regulating the proatheromatous T cell immune mechanism, the absence of PD-1 with PD-L1 can accelerate the formation of atherosclerotic plaque, and the absence of PD-1 receptor can prominently enhance the viability and factor secretion ability and fail to mediate external tolerance [17]. However, the PD-1/PD-L1 axis has a similar effect on the progress of AF which is unknown. In this work, the positive expression rate of PD-1 on CD4^+^ T lymphocytes in patients with AF was notably lower than the rate in control. Nevertheless, indifference was found in the expression level of CD8^+^ T lymphocytes. For its ligand, only the positive rate of PD-L1 on mDCs in the AF group was markedly decreased. Previous studies have identified that the content of PD-L1 on T lymphocytes may modulate the cell's own PD-1/PD-L1 axis, but the expression of PD-L2 is limited [18]. This study found no difference in the positive rate of PD-L2 on mDCs and the positive expression rates of PD-L1 and PD-L2 on CD4+ and CD8+ T cells, which further confirmed that PD-L2 was presented in T lymphocytes and can be detected by streaming techniques. From the above results, it can be concluded the PD-1 together with PD-L1 may be related to immune modulation of AF through T lymphocytes and mDCs cells.

T lymphocytes are divided into different subgroups according to different secreted factors, among which Th1/Th2 subgroup dysregulation exerts an important function in various autoimmune diseases or inflammatory diseases [19]. The results of this study found that the concentrations of ILs and IFN-*γ* in AF patients were memorably larger than the level in control, suggesting an out-of-balance of CD4^+^ T cells in patients with AF, and various inflammatory factors may be associated with the immune response and regulation of AF. Furthermore, in this research, after the content of PD-L1 was promoted by IFN-*α*, the capacity of mDCs to stimulate T cells to excrete cytokines of IL-2, IL-10, and IFN-*γ* was reversed in patients with AF, suggesting that the secretion capacity of inflammatory cells is intimately correlative to the proliferation of T cells under the regulation of PD-1/PD-L1 signaling pathway. Inflammatory responses characterized by T cell excitation participated in the modulation of the occurrence and progress of AF. Multivariate COX analysis demonstrated that patients with large SIS scores exhibited an increased risk of stroke. The KM survival curve showed that the stroke risk of high-level SIS was higher than that of low-level and intermediate-level patients (*P* < 0.05). This shows that peripheral inflammatory factors are risk factors for increased stroke risk in patients with AF, and the AF stroke risk enhances with increase of SIS score. Analysis of the relevant reasons may be that patients with AF have an inflammatory response in the body, which can further activate the interaction with fibrinolytic or coagulation factors, promote the procoagulation and proinflammatory state of AF, and increase the risk of stroke or death in AF. The results provide a theoretical basis for the causal association between inflammatory factors and the prognosis of AF and have certain clinical value for evaluating the prognosis of AF.

In conclusion, the PD-1 and PD-L1 pathway may promote the excretion of cytokines by regulating T cell excitation and exert an immunoregulatory effect on the pathogenesis of AF. This study is the first to explore the relationship between inflammatory status and prognosis in patients with AF, providing some evidence for the relationship between AF and inflammation. However, there are still some limitations, such as the small sample size and the lack of long-term follow-up outcomes. In addition, the established inflammation score is relatively simple, convenient, and low-cost. Thus, it needs to be evaluated by further large-sample and long-term follow-up studies.

## Figures and Tables

**Table 1:** Comparison of general data between the two groups.

General data	AF (*n* = 45)	Control (*n* = 45)	*χ* ^2^/*t* value	*P* value
Gender (male/female)	26/19	28/17	0.185	0.667
Age (year)	63.25 ± 8.47	64.21 ± 7.42	0.572	0.569
Body mass index (kg/m^2^)	23.45 ± 4.18	22.52 ± 4.28	1.043	0.300
Total cholesterol (mM)	5.20 ± 1.47	4.92 ± 0.55	1.197	0.234
LDL cholesterol (mM)	3.01 ± 0.75	2.98 ± 0.50	0.233	0.824
Leukocyte (10^9^/L)	6.11 ± 1.02	5.94 ± 0.70	0.922	0.359

**Table 2:** The expression changes of CD69 and HLA-DR on CD3+ T lymphocytes in external blood of two groups (*x* ± *s*).

Group	Cases	CD69	HLA-DR
AF	45	1.62 ± 0.41	36.62 ± 9.58
Control	45	1.02 ± 0.27	25.11 ± 8.39
*t* value		8.199	6.063
*P* value		<0.001	<0.001

**Table 3:** Changes of PD-1 expression on CD4+ and CD8+ T lymphocytes in external blood of two groups (*x* ± *s*).

Group	Cases	CD4^+^ PD-1	CD8^+^ PD-1
AF	45	1.82 ± 0.45	3.77 ± 0.29
Control	45	12.16 ± 1.75	3.62 ± 0.44
*t* value		38.387	1.910
*P* value		<0.001	0.060

**Table 4:** Changes of PD-L1 and PD-L2 expression on external blood mDC cells in two groups (*x* ± *s*).

Group	Cell	PD-L1	PD-L2
AF (*n* = 48)	mDCs	0.28 ± 0.10∗	1.32 ± 0.21
	CD4^+^	1.99 ± 0.28	1.25 ± 0.26
	CD8^+^	3.41 ± 0.87	0.95 ± 0.20
Control (*n* = 48)	mDCs	0.95 ± 0.31	1.40 ± 0.16
	CD4^+^	2.01 ± 0.39	1.30 ± 0.27
	CD8^+^	3.53 ± 1.08	1.02 ± 0.27

Note: Compared with the control group on mDC cells, ∗*P* < 0.05.

**Table 5:** Comparison of the expression of inflammatory factors in external blood between the two groups (*x* ± *s*).

Group	Cases	IL-2 (pg/ml)	IL-6 (pg/ml)	IL-10 (pg/ml)	IL-17A (pg/ml)	TNF (pg/ml)	IFN-*γ* (pg/ml)
AF	45	0.86 ± 0.51	6.13 ± 1.84	2.02 ± 0.24	9.44 ± 3.20	1.70 ± 0.25	2.39 ± 0.48
Control	45	0.15 ± 0.27	1.45 ± 0.26	1.33 ± 0.19	8.54 ± 2.16	1.66 ± 0.29	1.63 ± 0.29
*t* value		8.254	16.894	15.121	1.564	0.701	9.091
*P* value		<0.001	<0.001	<0.001	0.122	0.485	<0.001

**Table 6:** Effects of PD-1/PD-L1 signaling pathway on T lymphocyte immune function in patients with AF (*x* ± *s*).

Group	Cases	IL-2 (pg/ml)	IL-10 (pg/ml)	IFN-*γ* (pg/ml)
AF	45	3.25 ± 1.12	0.89 ± 0.10	12.51 ± 2.38
Control	45	2.11 ± 0.48	1.58 ± 0.23	9.45 ± 1.25
IFN-*α*	45	2.10 ± 0.26	1.60 ± 0.34	9.22 ± 2.80
*F* value		38.01	123.61	30.23
*P* value		<0.001	<0.001	<0.001

**Table 7:** Multivariate COX analysis of the relationship between external blood inflammatory factors and stroke in patients with AF.

Factors	*B*	*SE*	*Wald*	*P*	*OR*	95% CI
IL-2	0.189	0.058	9.916	0.002	1.208	1.012~1.459
IL-6	0.420	0.116	12.015	<0.001	1.508	1.221~1.748
IL-10	0.416	0.107	10.512	<0.001	1.622	1.132~1.879
IL-17A	0.518	0.275	3.142	0.078	1.663	0.958~2.745
TNF	0.259	0.155	2.675	0.125	1.385	1.235~1.758
IFN-*γ*	0.116	0.112	4.525	0.021	1.185	1.021~1.528

**Table 8:** Comparison of SIS scores between the two groups with and without stroke.

SIS	Stroke		No stroke	
	Cases	Percent	Cases	Percent
≤2	1	25.00	27	65.85
3~4	1	25.00	11	26.83
≥5	2	50.00	3	7.32
Total	4	100.00	41	

**Table 9:** Multivariate COX analysis of the relationship between SIS score and stroke in patients with AF.

Factors	*B*	*SE*	*Wald*	*P*	*OR*	95% CI
SIS≤2	0.258	0.175	2.676	0.102	1.598	0.902~1.780
SIS3~4	0.558	0.254	3.042	0.084	1.883	0.849~2.1564
SIS≥5	0.415	0.192	8.847	0.003	1.548	1.126~1.990

## Data Availability

The data used to support the findings of this study are included within the article.
